# A comparative analysis of the clinicopathological profile of early-onset versus late-onset rectal cancer patients

**DOI:** 10.3332/ecancer.2022.1365

**Published:** 2022-03-14

**Authors:** Vinita Trivedi, Richa Chauhan, Santosh Subham, Rita Rani, Usha Singh

**Affiliations:** Department of Radiation Oncology, Mahavir Cancer Sansthan, Patna 801505, India

**Keywords:** rectal cancer, young patients, grade, stage

## Abstract

**Introduction:**

Colorectal cancer has been primarily considered a disease of the elderly, but recent data have shown an alarming rise among young people. It has been also suggested that young age is associated with aggressive histopathological characteristics and advanced stages of the disease at diagnosis. As there are few studies and none from our part of the country evaluating the clinicopathological profile of early-onset versus late-onset rectal cancer patients, this analysis was conducted to assess and compare the clinical and pathological characteristics of patients with rectal cancer diagnosed with ages over and below 50 years.

**Materials and method:**

The relevant details of all biopsy proven rectal cancer patients undergoing radiotherapy at a tertiary cancer hospital, from January 2017 to December 2019, were collected. All the data were categorised into two groups, an early-onset group (age <50 years) and a late-onset group (age ≥50 years), and comparison of the clinicopathological characteristics between the two groups was made.

**Results:**

A total of 224 patients with rectal cancer, 150 male and 74 female, were included in the study. About two-thirds of the patients were less than 50 years of age, with an average age of 42 years. The comparative analysis showed a significantly higher number of young patients presenting with bleeding and pain. Patients below 50 years also had a significantly higher number of adenocarcinoma grade III and clinical stage III than those in the late-onset group.

**Conclusion:**

Our study revealed a significant number of early-onset rectal cancer patients. There should be a high index of suspicion in any young patient presenting with symptoms suggestive of rectal malignancy and they should be evaluated promptly.

## Introduction

According to the GLOBOCAN 2020 data, colorectal cancer (CRC) is the third most commonly diagnosed cancer in recent years, and the second leading cause of cancer deaths globally. With 19.29 million new cases and 9.96 million deaths, it accounts for 10% of the global cancer incidence and 9.4% of cancer deaths [1]. Rectal cancer alone accounts for 0.7 million new cases and is predicted to rise to 1.16 million by 2040. Rectal cancer incidence rates show a varied geographical distribution with high age-standardized rate (ASR) of 16.9 in males and 8.9 in females of Eastern Europe as compared to only 2.8 in males and 1.9 in females of South and Central Asian countries, including India [1]. In India, breast cancer is the most prevalent cancer with an incidence of 13.5%, followed by oral cavity (10.3%) and cervical cancers (9.4%). Although ranked 16th by incidence rate, India reported about 0.06 million new cases with 0.039 million deaths due to rectal cancer in 2020, which is further estimated to rise to 0.11 million new cases and 0.064 million deaths in 2040 [2]. As per the recent NCDIR report, which consolidates the data collected during the period 2012–2019 across 96 hospital-based cancer registries, from different parts of India, gastrointestinal (GI) cancers comprised 18% of overall cancer cases in the country. Among GI cancers, rectal cancer was the third most common cancer after oesophagus and stomach with an overall incidence rate of 2.6%. However, in the younger age group (20–34 years) with GI cancers, rectal cancer comprised over a quarter of the patients [3]. CRC usually begins with the non-cancerous proliferation of mucosal epithelial cells, which gradually evolve into pre-neoplastic lesions, such as low- and high-grade dysplasia, tubular and villous adenoma, and into carcinoma over years [4]. The cancer arises when certain cells of the epithelium acquire a series of genetic or epigenetic mutations that selectively increases their replication and survival [5]. The cause could be genetic, but sporadic cases have been steadily rising worldwide, especially in developing countries that are adopting the ‘western’ way of life [6, 7]. Certain dietary and lifestyle factors, like obesity, sedentary lifestyle, red meat consumption, alcohol and tobacco, can promote intestinal inflammation and modify the intestinal microflora to promote an immune response, both of which can facilitate the process of carcinogenesis [8]. The human intestinal microbiota is composed of 10^13^–10^14^ microbes, which can regulate the initiation and progression of CRC by producing genotoxins, metabolites from dietary components, biofilms and oxidative stress [9, 10]. Intestinal microbiota can be used as a biomarker for screening and as a prognostic marker [11]. Furthermore, it can be modulated for prevention and treatment of CRC [12].

Early detection and removal of these precancerous lesions by colonoscopy lead to a significant reduction in CRC incidence and mortality [13]. CRC has been primarily considered a disease of the elderly, which mostly occur after the fifth decade of life [14]. Therefore, CRC screening has been recommended by various advisory committees across the world from ages 50 through 75 [15]. However, recent data from Western and Asian countries have shown an increase in the number of cases among patients under the screening age of 50 [16, 17]. As a result, the US Preventive Services Task Force has issued new guidelines recently that recommend colon cancer screening start at the age of 45 instead of 50 [18]. Furthermore, studies also suggest that young age is associated with more advanced stages of the disease at diagnosis and with more aggressive histopathological characteristics leading to poor survival [19]. However, few authors contradict these findings, describing results similar to those of patients with a later diagnosis, or even with improvement in their survival [20, 21]. The prognosis of the younger CRC patients is an important issue due to the impact of the disease and its treatment strategies on their fertility, life expectancy and quality of life [22].

In recent years, there has been a general perception among oncologists in India that most cases of CRC present at a younger age, with an aggressive histopathology, and with involvement of both rectum and anal canal [23]. India is a large country with a variety of ethnic groups, cultures, environments, foods and lifestyles, and thus exhibits a wide heterogeneity in the geographic incidence of cancer. There have been a few studies conducted in different parts of India describing the epidemiological and clinical profiles of rectal cancer patients, but there has been no such study from our state of Bihar, the second largest state by population with close to 125 million people. We believe that simple descriptive studies provide real-world data which may help in understanding the disease and adopting strategies to reduce the burden, morbidity and mortality of the disease in developing countries. Hence, this study was conducted to assess and compare the clinical and pathological characteristics of patients with rectal cancer diagnosed with ages over and below 50 years.

## Materials and methods

From 1 January 2017 to 31 December 2019, all consecutive rectal cancer patients who were treated in the Department of Radiotherapy, Mahavir Cancer Sansthan, Patna, were evaluated for this retrospective study. Since this is a retrospective study, approval from the Institutional Ethics Committee was not required as part of our institutional protocol, and the need for obtaining written informed consent was also waived. The study included all biopsy-proven rectal carcinoma patients treated with radiotherapy during the study period. Patients receiving previous chemotherapy and/or radiotherapy and those with histology other than carcinoma were excluded from the study cohort. Medical records of these patients were analysed for the required data. Parameters studied included age, sex, site of lesion, clinical presentations, duration of symptoms, histopathology of the lesion, stage of the disease, need for colostomy and intent of radiotherapy. The histological subtypes and grades of carcinoma were assigned based on the WHO’s classification. The anatomical location was defined as rectum (disease extending from anorectal ring to rectosigmoid junction), rectum and anal canal (bulk of the disease in the rectum and also involving anal canal) and rectum and sigmoid colon (bulk of the disease in the rectum and also involving part of sigmoid colon). The cancer stage at the initial diagnosis was defined according to the 7th edition of American Joint Committee on Cancer TNM staging system. The staging was based on findings from proctoscopy, colonoscopy, chest X-ray, computed tomography (CECT), magnetic resonance imaging (MRI) and positron emission tomography as available.

### Statistical analysis

All the available data were entered into a Microsoft excel sheet. Categorical variables were summarised by descriptive statistics using percentage. The patients were further divided into two groups, an early-onset group (age <50 years) and a late-onset group (age ≥50 years), for comparing clinicopathological characteristics between the two groups. Chi-square test was used to evaluate the differences in percentages between the early-onset group and the late-onset group. A *p*-value < 0.05 was considered statistically significant.

## Results

**Figure 1.**
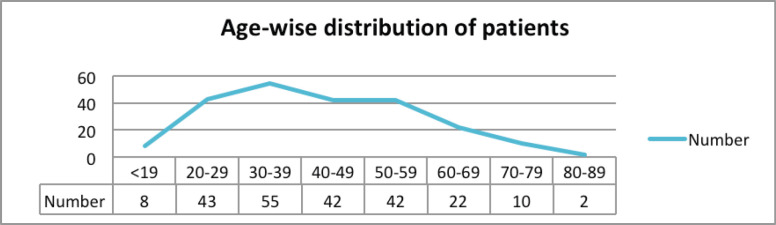
Age-wise distribution of rectal cancer patients.

A total of 224 patients with rectal cancer were included in the study. Among 224 patients, 150 (69.73%) were male and 74 (33.03%) were female. The average age at diagnosis was 42 years with a range from 13 to 87 years. The most common age group seen was 30–39 years with 24.55% patients. About two-thirds of the patients were less than 50 years of age (Table 1; Figure 1).

**Figure 2.**
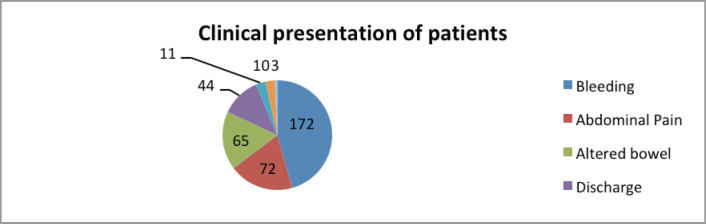
Clinical presentation of rectal cancer patients.

Majority of the patients (129, 57.58%) had a low-lying rectal cancer with anal canal involvement. In 70 (31.25%) patients, the disease was confined to the rectum. The remaining 25 (11.16%) patients had rectal cancer involving the sigmoid colon. The most common presenting symptom was bleeding per rectum, followed by pain, altered bowel habit, mucous discharge per rectum, and faecal incontinence seen in 76.78%, 32.14%, 29.01%, 18.33% and 4.91% of the patients, respectively. In addition, 4.46% of the patients presented with intestinal obstruction and 1.33% with intestinal perforation. Diversion colostomy was required in 36 (16.07%) patients. The average duration of presenting complaints was 8.94 months. Among the patients, 44.64% had symptoms for 3–6 months, 29.46% for 6 months to 1 year and 9.82% for more than 1 year (Table 2; Figure 2).

**Figure 3.**
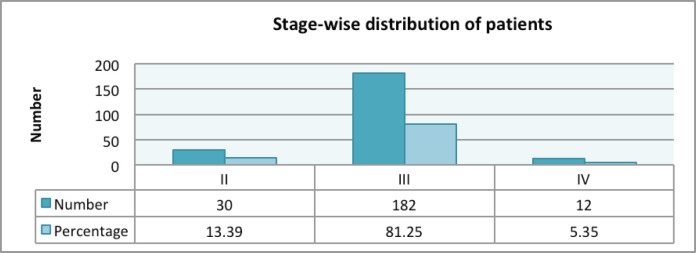
Clinical stage of rectal cancer patients.

Adenocarcinoma was the most common histology seen in 99% of the patients, with only two patients having squamous cell carcinoma. Most of the patients (40.62%) had a grade II tumour, followed by grade I and III tumours in 29.91% and 29.46% of the patients, respectively. Signet ring cell carcinoma and mucinous carcinoma were present in 3.12% and 0.89% of the patients, respectively (Table 3). Stage III was the most common presenting stage in 182 (81.25%) patients, followed by 30 (13.39%) and 12 (5.35%) patients with stage II and IV, respectively (Figure 3). In patients with stage IV disease, the most common site of distant metastases was liver, followed by peritoneal, bone and lung. Liver metastases were seen in seven patients, with two of them also having peritoneal deposits and one having lung metastases. Peritoneal deposit was the only site of metastases in two patients. Bone metastases were reported in two patients and remaining one patient had lung metastases.

Radiotherapy was delivered with neoadjuvant intent in 194 (86.60%) patients. Post-operative radiotherapy was used in 19 (8.48%) patients and 11 (4.91%) patients were treated with a palliative intent (Table 4).

**Figure 4.**
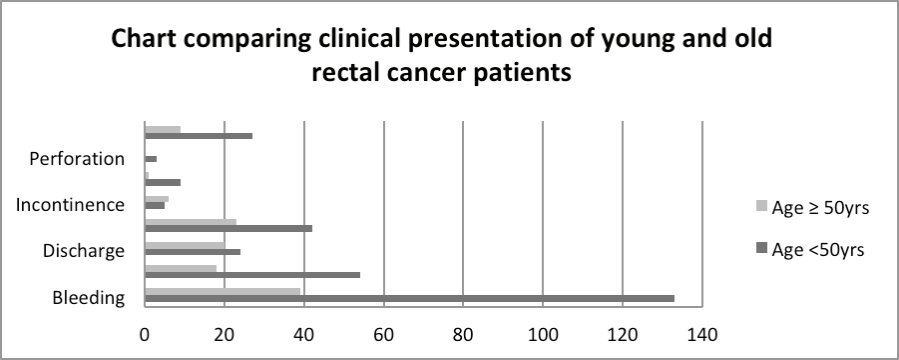
Comparative clinical presentation of young and old rectal cancer patients.

A comparative analysis of young onset rectal cancer patients (age <50 years) versus older rectal cancer patients (age ≥50 years) showed a slightly higher male preponderance in the older age group. Around 60% of the patients in the younger age group had the disease in the lower rectum involving anal canal as compared to 54% in the older age group, but the difference was not statistically significant (*p-*value = 0.43). Bleeding was reported in 89.86% of the patients in the younger age as compared to only 51.31% in the older age and this difference was statistically significant (*p*-value < 0.0001). Similarly, more patients in the younger age group complained of pain than older patients (36.48% versus 23.68%; *p*-value = 0.05). Intestinal obstruction and perforation were more commonly found in the younger age group as compared to faecal incontinence, which was more common in the older age group of patients, although the difference was not statistically significant (Figure 4). More patients (18.24%) in the younger age group required diversion colostomy as compared to 11.84% in the older age group. The average duration of presenting with complaints was 9.36 months in the younger age group as compared to 8.13 months in the older age group, but this difference was not statistically significant (*p*-value = 0.38) (Table 5).

Adenocarcinoma was the predominant histology seen in both the groups. However, young rectal cancer patients had a significantly higher incidence (36.48%) of poorly differentiated or grade III carcinoma as compared to 15.78% in the older age group (*p*-value = 0.001). Similarly, young patients reported a higher incidence of adenocarcinoma with signet ring component and signet cell carcinoma, which was 8.78% and 4.05%, as compared to 3.94% and 1.31% in the older age group, respectively. Furthermore, adenocarcinoma with mucinous component was reported in 6.08% of young patients as compared to 3.94% in the older age group (*p*-value = 0.50).

**Figure 5.**
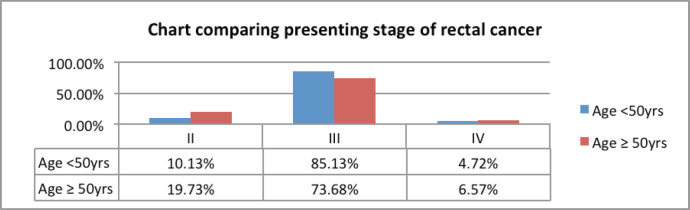
Comparative clinical stage at presentation of young and old rectal cancer patients.

About 85% of the young patients had a stage III disease as compared to 73.68% in the older age group (*p*-value = 0.03). Distant metastasis at presentation was slightly more common among older patients, which was 6.57%, as compared to 4.72% in the younger age group (*p*-value = 0.56) (Figure 5). Radiotherapy was delivered as a preoperative treatment to downstage the disease in 88.51% of young patients as compared to 82.89% of older patients. Furthermore, 11.84% of older patients had an upfront surgery and received adjuvant radiotherapy as compared to only 6.75% of patients in the younger age group (*p*-value = 0.19) (Table 6).

## Discussion

CRC is a major cause of morbidity and mortality worldwide with wide geographical variation in incidence and clinical presentation [24]. This study investigated the clinicopathological characteristics of rectal cancer patients according to the age of presentation. Our study population showed a male predominance with a male to female ratio of 2:1. This is in accordance to other studies reporting a higher incidence rate of CRC among males than females [17]. The male-to-female incidence rate ratio increases progressively across the colon from the caecum to the rectum from close to unity for cecal cancers to two for rectal cancers [25]. Although the reason for this is not completely understood, a study by Murphy *et al* [26] suggests that differential exposure to dietary and lifestyle-related risk factors like alcohol, consumption of red meat as well as a differential expression of hormonal and other receptors across the length of the colon and rectum could be the probable cause.

CRC has been long considered a disease of the elderly [27]. However, in our study cohort, the mean age of patients was 42 years and two-thirds of the patients were less than 50 years of age. Similar to our finding, various recent studies have reported that the incidence of CRC is increasing among young individuals in the Middle East and other regions in the world [28, 29]. A study from central India, on 233 patients over 8 years, reported the median age at diagnosis to be 43 years [30]. Another study from eastern India reported 47.01 years to be the mean age of presentation for patients with CRC [31]. A single-centre audit of CRC in India by Patil *et al* [23] concluded that CRC in India differs from that described in the Western countries and we have a higher proportion of young patients. Various studies have reported that CRC in young patients is more likely to have poor histological features and present in an advanced stage than in the older age group [32, 33]. These findings do suggest that CRC in young patients could be a different biological entity requiring aggressive treatment [34, 35]. However, there is a controversy over the effects of age on the presentation and survival of CRC patients. The cut-off ages 30, 35, 40, 45, and 50 years have been used in different studies [36–39]. We chose the cut-off age of 50 for the current study as it is the recommended age for CRC screening in the general population [40].

Regarding subsite distribution, low-lying rectal cancer involving anal canal was the most common site seen in our patients. Patil *et al* [23] from India also reported that 54% of the patients had an anorectal/rectal disease. Laskar *et al* [41] also reported a predominance of low rectal tumours in their study from patients in north-east India.

The most common presenting symptoms in these patients include pain, bleeding, discharge, altered bowel and obstructive symptoms [42]. In few cases, they may present in an emergency setting with intestinal obstruction or perforation. Similarly, the commonest symptom was rectal bleeding (89.86%), followed by pain (36.48%) and altered bowel habits (28.37%) in our patients. The presence of rectal bleeding and pain was significantly higher in the younger age group. Intestinal obstruction, perforation and colostomy were also more commonly reported in the younger age group. This could be because of the more aggressive nature of the disease in young patients or a relatively longer duration of symptoms [31, 43]. The average duration of presenting complaints was 9.36 months in the younger age group as compared to 8.13 months in the older age group. Furthermore, 86% of the patients in the younger age group complained of more than 3 months of duration of presenting complaints as compared to 80% in the older age group. The duration of presenting complaint in our study was much longer than that reported by Patil *et al* [23] in their audit on colorectal patients in India.

Our study also reported a significantly higher number of young CRC patients presenting with stage III disease as compared to the older patients. A higher number of advanced cancers at presentation could be attributed to the lack of population-based screening and timely access to healthcare [44] and health community coverage. There is also the possibility of delayed diagnosis, especially in younger patients where there is lesser suspicion of a malignancy due to assumed low incidence [45]. Most of the patients in the younger age group are initially treated for amoebiasis, granulomatous infections and worm infestations in our scenario, resulting in delay in diagnosis and treatment [46]. Many studies from India have also reported stage III as the most common presenting stage of the disease [23, 44]. The fact that most early-stage CRCs are asymptomatic and usually diagnosed at the time of screening, which is not very prevalent in our country, could also be the reason for advanced stage presentation in our study.

In our study, adenocarcinoma was the predominant histology in both the groups as has been reported in other studies [22, 41, 42]. The younger age group had a significantly higher percentage of poorly differentiated tumours than the older age group. A study by Ghodssi-Ghassemabadi *et al* [20] also concluded that younger patients had a significantly poor tumour grade than the older ones. Other studies have also reported higher prevalence of poorly differentiated signet cell histology in young colorectal patients suggesting an aggressive tumour [47]. Although statistically not so significant, younger patients showed a higher incidence of adenocarcinoma with signet ring component and signet ring cell carcinoma along with adenocarcinoma with mucinous component.

The intent of treating with radiotherapy was neoadjuvant in 88.51% of young patients as compared to 82.89% of older patients. Neoadjuvant therapy comprises a combination of radiotherapy and chemotherapy and is used to downsize or downstage the tumour in anticipation of surgical resection. In rectal cancer involving the anal sphincters, neoadjuvant therapy can potentially downsize a tumour to allow for the creation of a safe resection margin, thereby preserving the anal sphincters and maintaining anal continence [48]. Radiotherapy has been established as a mainstay of treatment alongside surgery in locally advanced rectal cancer and provides good symptomatic relief in these patients [49]. An analysis by the National Cancer Database in 2017 showed a pathologic complete response rate of 13% in an overall patient cohort of 27,532 receiving neoadjuvant therapy [50].

Before we conclude, it is important to describe the limitations of this study. Being a retrospective analysis, only documented details were available for evaluation. Many relevant data like details on adenoma, family history and Human Papilloma Virus (HPV) status were not available. As it was a monocentric study with data from a single department, the sample size was small. Many of the patients did not undergo staging MRI scan, so the exact T stage was not available for many patients. Furthermore, genetic testing was not conducted in these patients as our centre does not have genetic testing facility. Nonetheless, this study provides relevant data regarding the clinicopathological profile of rectal cancer patients and will add to the existing regional and world database for making valid conclusions.

## Conclusion

Our study revealed a significant number of young patients among rectal cancer patients. The younger age group had more patients presenting with an advanced stage and with poorly differentiated histology. Rectal bleeding in any patient should not be ignored but evaluated further with at least a digital rectal examination and a sigmoidoscopy. Early diagnosis and adequate treatment of young adults with CRC represent an unmet clinical need. There should be a high index of suspicion in any young patient presenting with symptoms suggestive of rectal malignancy and they should be evaluated promptly.

## Conflicts of interest

There is no conflict of interest among the authors.

## Funding

There is no funding for this study.

## Figures and Tables

**Table 1.** Gender and age distribution of rectal cancer patients.

Parameter	Number (*N* = 224)	Percentage
**Gender**		
Male	150	66.96
Female	74	33.03
**Age group (years)**		
10–19	8	3.57
20–29	43	19.19
30–39	55	24.55
40–49	42	18.75
50–59	42	18.75
60–69	22	9.82
70–79	10	4.46
80–89	2	0.88

**Table 2.** The clinical profile of rectal cancer patients.

Parameter	Number (*N* = 224)	Percentage
**Site**		
Rectum and anal canal	129	57.58
Rectum only	70	31.25
Rectum and sigmoid colon	25	11.16
**Symptoms**		
Bleeding	172	76.78
Abdominal pain	72	32.14
Altered bowel	65	29.01
Discharge	44	18.33
Incontinence	11	4.91
Obstruction	10	4.46
Perforation	3	1.33
Colostomy	36	16.07
**Duration of symptoms**		
Mean	8.94 months ± 1.31	
<3 months	36	16.07
3–6 months	100	44.64
6–12 months	66	29.46
>12 months	22 (9.82)	9.82

**Table 3.** The histopathological profile of rectal cancer patients.

Parameter	Number (*N* = 224)	Percentage
**Histology**		
SCC	2	0.89
Conventional adenocarcinoma	185	82.59
Adenocarcinoma with signet ring cell component	16	7.14
Signet ring cell carcinoma	7	3.13
Adenocarcinoma with mucinous component	12	5.36
Mucinous carcinoma	2	0.89
**Grade**		
I	67	29.91
II	91	40.62
III	66	29.46

**Table 4.** The presenting stage and intent of radiation in rectal cancer patients.

Parameter	Number (*N* = 224)	Percentage
**Stage**		
II	30	13.39
III	182	81.25
IV	12	5.35
**Intent of radiation**		
Neoadjuvant	194	86.60
Adjuvant	19	8.48
Palliative	11	4.91

**Table 5.** Comparative analysis of gender, site and clinical features of young and old rectal cancer patients.

Parameters	Age < 50 years (*N* = 148)	Age ≥ 50 years (*N* = 76)	Difference	*p*-value
**Gender**				
Male	97 (65.54%)	53 (69.73%)	4.19	0.53
Female	51 (34.45%)	23 (30.26%)	4.19	0.53
**Site**				
Rectum and sigmoid colon	45 (30.40%)	25 (32.89%)	2.49	0.70
Rectum	15 (10.13%)	10 (13.15%)	5.65	0.21
Rectum and anal canal	88 (59.45%)	41 (53.94%)	5.51	0.43
**Symptoms**				
Bleeding	133 (89.86%)	39 (51.31%)	38.55	<0.0001
Abdominal pain	54 (36.48%)	18 (23.68%)	12.80	0.05
Discharge	24 (16.21%)	20 (26.31%)	10.10	0.07
Altered bowel	42 (28.37%)	23 (30.26%)	1.89	0.76
Incontinence	5 (3.37%)	6 (7.89%)	4.52	0.13
Obstruction	9 (6.08%)	1 (1.31%)	4.77	0.10
Perforation	3 (2.02%)	0	2.02	0.21
Colostomy	27 (18.24%)	9 (11.84%)	6.40	0.21
**Duration of symptoms**				
Mean	9.36 months ± 1.67	8.13 months ± 2.08	1.23	0.38
<3 months	21 (14.18%)	15 (19.73%)	5.55	0.28
3–6 months	67 (45.27%)	33 (43.42%)	1.85	0.79
6–12 months	44 (29.72%)	22 (28.94%)	0.78	0.90
>12 months	16 (10.81%)	6 (7.89%)	2.92	0.48

**Table 6.** Comparative analysis of pathology, clinical stage and intent of radiation in young and old rectal cancer patients.

Parameters	Age < 50 years (*N* = 148)	Age ≥ 50 (*N* = 76)	Difference	*p*-value
**Histology**				
SCC	2 (1.35%)	–	1.35	0.31
Conventional adenocarcinoma	119 (80.40%)	68 (89.47%)	9.07	0.08
Adenocarcinoma with signet ring cell component	13 (8.78%)	3 (3.94%)	4.84	0.18
Signet ring cell carcinoma	6 (4.05%)	1 (1.31%)	2.74	0.26
Adenocarcinoma with mucinous component	9 (6.08%)	3 (3.94%)	2.14	0.50
Mucinous carcinoma	1 (0.89%)	1 (1.31%)	0.42	0.47
**Grade**				
I	42 (28.37%)	25 (32.89%)	4.52	0.48
II	52 (35.13%)	39 (51.31%)	16.18	0.01
III	54 (36.48%)	12 (15.78%)	20.70	0.001
**Stage**				
II	15 (10.13%)	15 (19.73%)	9.60	0.04
III	126 (85.13%)	56 (73.68%)	11.45	0.03
IV	7 (4.72%)	5 (6.57%)	1.85	0.56
**Intent of radiation**				
Neoadjuvant	131 (88.51%)	63 (82.89%)	5.62	0.24
Adjuvant	10 (6.75%)	9 (11.84%)	5.09	0.19
Palliative	7 (4.72%)	4 (5.26%)	0.54	0.85
